# High power downconversion deep-red emission from Ho^3+^-doped fiber lasers

**DOI:** 10.1515/nanoph-2021-0763

**Published:** 2022-03-14

**Authors:** Shuaihao Ji, Yingyi Song, Zhongyu Wang, Chenyao Shen, Jiazhen Lin, Bo Xiao, Qichen Feng, Qingyang Du, Huiying Xu, Zhiping Cai

**Affiliations:** Department of Electronic Engineering, Xiamen University, Xiamen, 361005, China

**Keywords:** deep-red laser, high power, Ho^3+^-doped fiber

## Abstract

It has been a big challenge in decades for Ho^3+^-doped fiber laser to produce high emission power in deep-red due to its intrinsic long carrier lifetime in ^5^I_7_ energy level. Here, we advance the development of this visible fiber lasers by a novel pumping method that leverages the advantages of blue laser diode downconversion mechanisms to fully pumping, while shortening the lifetime of the lower energy level of the deep-red laser through excited state absorption. For the first time, we demonstrate a downconversion fiber laser using a single wavelength of 532 nm solid-state laser pump source to excite the Ho^3+^-doped ZBLAN fiber, which achieves a record high maximum output power of 1.64 W at 752.10 nm, as well as free-running of deep-red lasers with watt-level output. This technology represents a milestone in high power deep-red fiber lasers. Importantly, it is readily to be extended to other wavelengths that evidenced its huge application potentials in fiber laser industry.

## 1 Introduction

High power fiber lasers have made important advances and applications in the near to mid-infrared region, relying on the mature industrial base of rare earth doped fibers, low attenuation of light and precise parameter control to achieve high pumping efficiency assurance. Fiber lasers in the 1–2.25 μm range can be generated directly or by nonlinear effects and produce output powers in excess of 100 W [1], [2], [3], [4], [5]. Visible fiber lasers are making rapid progress in many fields such as in biomedicine, fine processing, spectroscopy [6], [7], [8]. There are various technical approaches to the implementation of visible fiber lasers, which can be broadly classified into four categories: (I) visible lasers by using nonlinear optical crystals that multiply the fiber laser at infrared wavelengths [9], [10], [11]; (II) optical parametric oscillators based on fiber nonlinearity [12]; (III) upconversion fiber lasers doped with rare-earth ions [13, 14]; (IV) direct downconversion fiber lasers doped with rare-earth ions lasers [15, 16].

**Figure 1:**
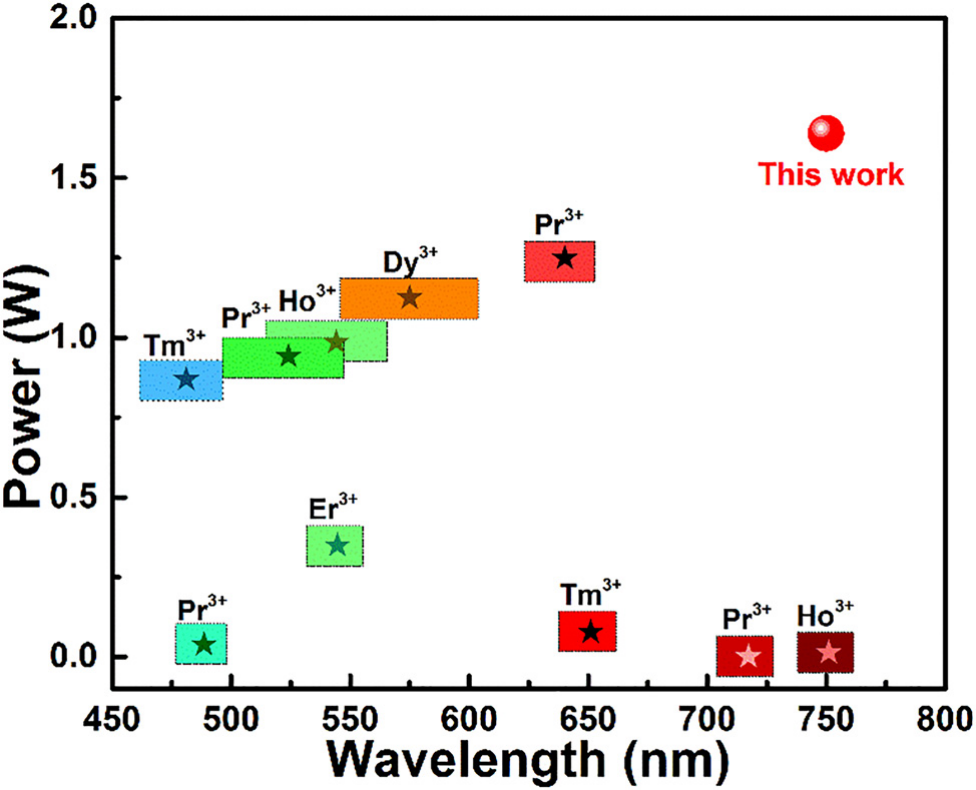
The output performances of visible fluoride glass fiber lasers reported to date [14, 18], [19], [20], [21], [22].

In order to directly generate lasers in the visible band, the rare-earth ions should be used as doping sources, such as Tm^3+^, Er^3+^, Pr^3+^, Dy^3+^, and Ho^3+^ ions [14, 17–22]. It is well known that most current visible lasers rely on fluoride glass for their implementation. However, due to the act that the thermal, physical, mechanical and chemical properties of fluoride glass fibers are far inferior to those of silicate glass fibers, the output performance of rare-earth doped fluoride visible fiber lasers has long been limited. The highest output power and luminescence wavelengths of single-cladding fluoride fibers doped with different rare-earth ions in the visible band and their spectral ranges are summarized in Figure 1. Watt-level fiber laser output has been achieved by different rare-earth ions doping in the visible wavelength band less than 650 nm. But there is still a large gap in the development of high power fiber lasers in the deep-red wavelength band. The main factors limiting the power scalability of visible lasers can be attributed to the low damage threshold of fluoride fibers on the one hand and the lack of a suitable pumping mechanism on the other hand.

Comparing the energy level structures of other rare-earth ions and their properties, the luminescent properties of Ho^3+^ in the visible band make it possible to generate deep-red light excitation. Ho^3+^-doped fluoride deep-red fiber lasers were first investigated in 1990 [23], using a krypton-ion laser with a wavelength of 647.1 nm as the pump source. Due to the pumping mechanism and pump power limitation, the output power was only 2 mW at 750 nm. Until the downconversion mechanism under the blue LD as the pump source was proposed in 2018 [24], a green fiber laser operating at 543 nm generated an output power of 150 mW. Unfortunately, the deep-red laser output properties were not considerably improved, with an output power of 16 mW and a slope efficiency of only 3.9%, one reason is that the available pump power of the blue LD coupled into the active fiber is limited, and the other is that the lower energy level ^5^I_7_ of the deep-red laser has a fluorescence lifetime longer than 10 ms [25]. Green light as a pump source to excite Ho^3+^-doped fibers is proven to be feasible and has been reported in the mid-infrared band [26, 27]. Commercial solid-state lasers operating at 532 nm have witnessed tremendous growth in recent years, with output power of up to 20 W coupled to multimode fiber [28]. Therefore, combining the advantages of high conversion efficiency of downconversion pumping and the shortening of excited state fluorescence lifetime by strong excited state absorption (ESA), we used a 532 nm solid-state laser as the pump source, which effectively addresses the issues caused by blue LD and red pumping and enables us to operate deep-red lasers with high power and high efficiency.

In this paper, we present a solid-state laser at 532 nm as a pump source to excite Ho^3+^-doped ZBLAN fibers to generate lasers in the visible range at deep-red wavelengths, for the first time. We have achieved an output power of 1.64 W at 752.10 nm, which is the highest output power in a single-cladding fiber in the visible band, and a slope efficiency of 50.2%. We further obtained watt-level free-running deep-red lasers without the need of additional laser cavity mirrors. By comparing the blue LD downconversion and red solid-state upconversion pumping mechanisms, we are convinced that this method introduces a new approach of achieving fiber laser output in the visible band in terms of improved laser output characteristics and increased energy conversion efficiency. This technology will be suitable for high-power green solid-state pumped double-cladding Ho:ZBLAN fibers that can be used to realize the scalable power improvement of deep-red lasers.

## 2 Theoretical analysis and experimental principle

### 2.1 Spectral properties and energy levels of Ho^3+^-doped ZBLAN fiber

Ground state absorption (GSA) spectra of active fiber was measured at room temperature by a HL-2000 Halogen light source (Ocean Optics) and by an optical spectrum analyzers (OSA, AQ6315B, Ando) with resolution of 0.05 nm. In our experiment, the active fiber was provided by a Ho^3+^-doped ZBLAN fiber from Le Verre Fluoré, France (core/cladding: 7.5/125 μm, NA: 0.23, dopant concentration: 5000 ppm). The calculation of the absorption cross section is based on Beer Lambert’s law [29], which is expressed as:

$$\sigma_{abs}=\frac{2.303\cdot \text{log}\left(I_{0}/I\right)}{NL}$$

Where *I*_0_ and *I* are the optical intensity of incident light and transmitted light, respectively. *N* is the doping concentration of rare earth ions in the fiber. For 0.5% mol Ho^3+^:ZBLAN, it has a value of 2.935 × 10^19^ cm^−3^. *L* is the fiber length.

**Figure 2:**
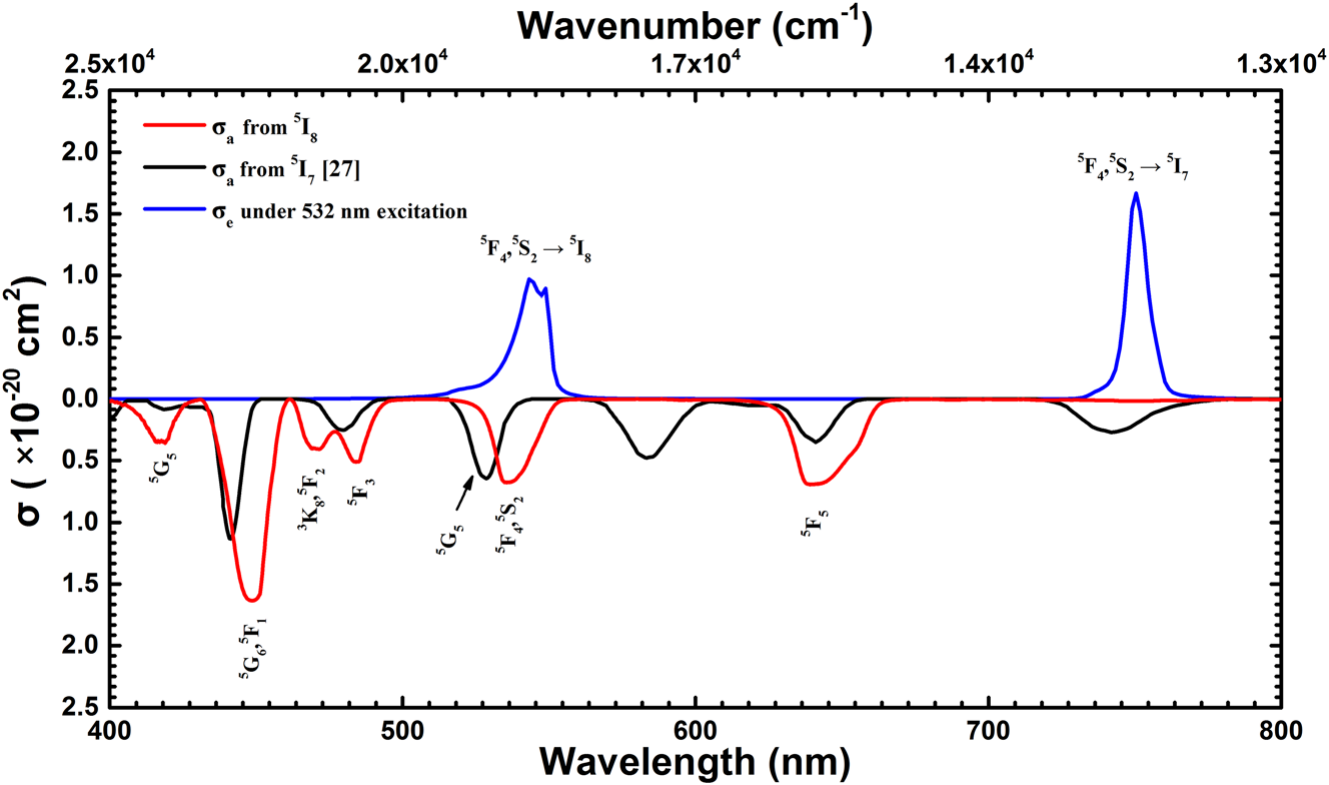
Spectra of GSA (red), ESA (black) and fluorescence emission (blue) cross sections along with assignments of Ho^3+^-doped ZBLAN fiber.

The GSA cross section of Ho^3+^-doped ZBLAN fiber in the visible to near-infrared band is plotted by the red line in Figure 2. At 532 nm, the absorption cross section is about 0.5 × 10^−20^ cm^2^. It is worth noting that 532 nm is not at the GSA peak in the green band. Interestingly, the excited state ^5^I_7_ absorption cross section represented by the black curve in Figure 2 is up to 0.5 × 10^−20^ cm^2^ [27]. As we all know, this will greatly shorten the lifetime of the ^5^I_7_, which is as long as 13.6 ms. Moreover, the lifetime of the lower level of the laser is an important factor restricting the laser output characteristics of the four level structure.

Emission spectra was measured by excitation with a commercial 532 nm continuous wave (CW) solid-state laser. According to the Fuchtbauer–Ladenburg theory, the emission cross section can be deduced from the emission spectrum of the relevant transition [30], and it can be written as follows:

$$\sigma_{e}\left(v\right)=\frac{1}{\tau }\cdot \frac{\lambda^{4}I\left(\lambda \right)}{8\pi n^{2}c\int I\left(\lambda \right)d\lambda }$$

Where, *λ* is the center wavelength, and *I*(*λ*) is the corresponding emission spectrum intensity. *n* is the refractive index of the core, and *c* is the speed of light. Here *τ* represents the fluorescent lifetime for related energy level.

The blue curve in Figure 2 shows the emission cross section of Ho^3+^-doped ZBLAN fiber in the visible band. As typical spectral resources, the emission cross sections of green (543 nm) and deep-red (750 nm) are 1.0 × 10^−20^ cm^2^ and 1.7 × 10^−20^ cm^2^, respectively. In view of the reabsorption effect of Ho^3+^-doped ZBLAN fiber in green band, and the strong ESA of ^5^I_7_ at 532 nm, this paper mainly presents a deep-red downconversion fiber laser with 532 nm as the pump source.

**Figure 3:**
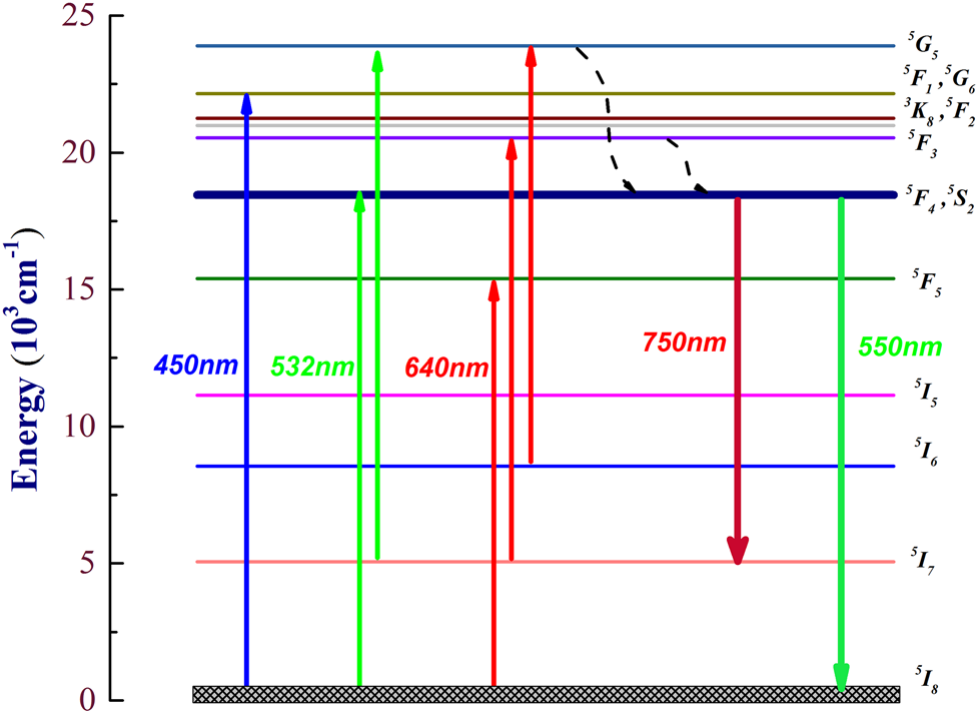
Partial energy-level diagram of Ho^3+^-doped ZBLAN fiber pumped by a 532 nm solid-state laser.

The downconversion pumping process with a 532 nm pump is shown in Figure 3. First, ground state (^5^I_8_) ions are excited to the higher state level and decay to the metastable ^5^F_4_, ^5^S_2_ level by multiphonon relaxation. These ions are re-excited to the ^5^G_5_ level by ESA and decay to the ^5^F_4_, ^5^S_2_ level. This process reduces the population of ^5^I_7_ and increases the population of ^5^F_4_, ^5^S_2_.

Compared with our previous work [22, 24], the ground state particles are pumped to a higher excited state ^5^G_6_, ^5^F_1_ under the blue LD 450 nm pump downconversion, while the two-photon sequential absorption upconversion process under the red light 640 nm pump also causes energy loss due to multi-phonon relaxation transition. In this paper, a green light 532 nm solid-state laser was used as the pump source to significantly reduce the energy loss caused by non-radiative transitions, providing higher energy conversion efficiency. More importantly, due to the strong ESA of ^5^I_7_ for 532 nm pump, the effective energy level equivalent lifetime is greatly reduced, which creates a new method to produce high-performance deep-red laser.

### 2.2 Experimental setup

**Figure 4:**
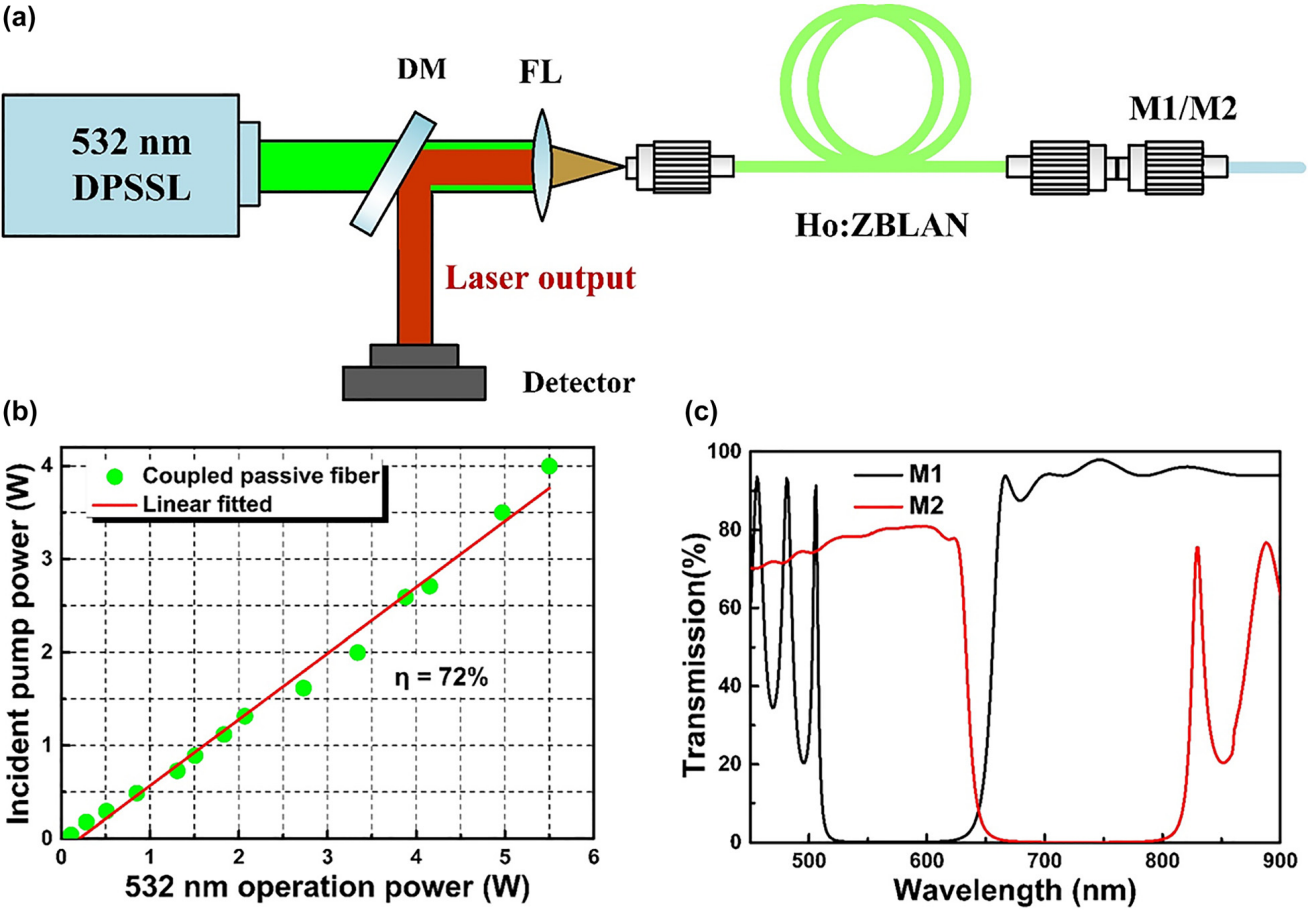
The configuration of deep-red fiber lasers. (a) Schematic of the experimental setup for the Ho^3+^-doped ZBLAN visible fiber laser. (b) Incident pump power as a function of the 532 nm solid-state laser output. (c) Optical transmission spectra of the fiber end-facet mirror (M1/M2).

Figure 4(a) shows the experimental setup of the Ho^3+^-doped ZBLAN visible fiber laser. Such a visible oscillation consists of a fiber end-facet mirror (i.e., M1 or M2) and a series of active fibers of different lengths. The pump source is a commercial diode-pumped solid-state laser based on frequency doubling technology operating at 532 nm. As presented in Figure 4(b), the green pump source has a maximum fundamental output power of 6 W and coupled into a passive fiber (core/cladding: 7.5/125 μm, NA: 0.26), in correspondence to a slope efficiency of 72%. Figure 4(c) gives the optical transmission properties of the fiber end-facet mirrors, which were designed with a high reflectivity at signal wavelengths (i.e., M1: *R* = ∼99.95%@∼543 nm; M2: *R* = ∼99.95%@∼750 nm). It is worth noting that the M1 mirror is specially designed to have a transmission of more than 90% in the deep-red band, while the M2 mirror also increases the transmission in the green band as much as possible. In combination with our description of the emission cross section of the Ho^3+^-doped ZBLAN fiber in the visible band in Figure 2, high-quality coating of the fiber end facets were required to ensure the single wavelength output of the target laser.

## 3 Results and discussion

### 3.1 High output performance of deep-red fiber laser

**Figure 5:**
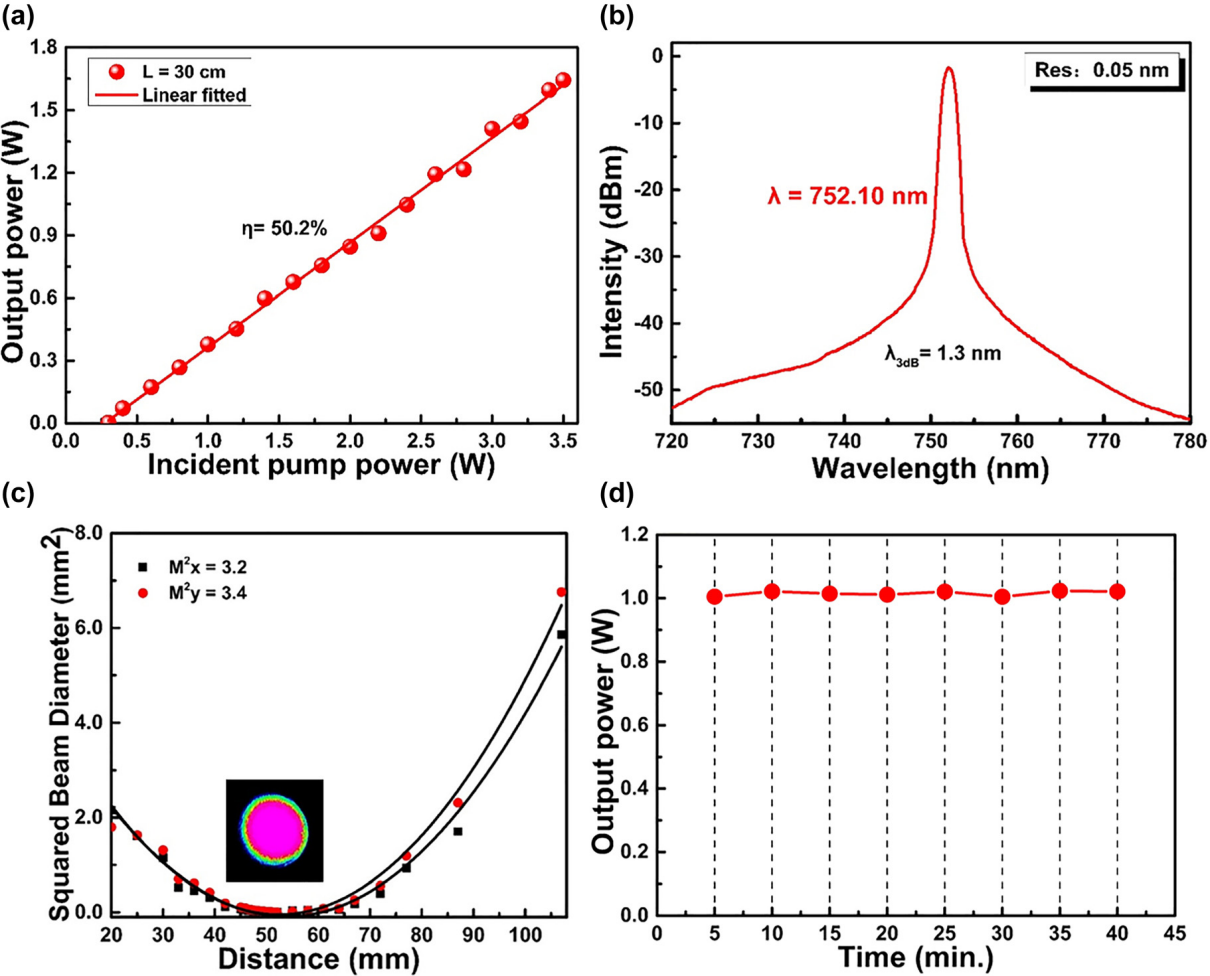
High performances of deep-red fiber laser with a 30 cm Ho^3+^-doped ZBLAN fiber. (a) The output power as a function of the incident pump power. (b) The optical spectra under the maximum output power. Measured (c) beam quality *M* ^2^ and (d) power stability at ∼1 W of deep-red output power.

Figure 5 exemplifies the lasing characteristics of high performance deep-red fiber laser oscillation for the active fiber length of 30 cm. As mirrors in the laser resonator, M2 has a high reflectivity of >99.9% in the deep-red oscillation wavelength range, and the end facet of the Ho^3+^-doped fiber with a ∼4% Fresnel reflection was used as the laser output. The output power characteristic as a function of the incident pump power is presented in Figure 5(a). The slope efficiency with respect to the incident pump power was 50.2% with an oscillation threshold of 0.30 W. The maximum output power achieved is 1.64 W, which is the highest output power from Ho^3+^-doped fiber lasers in the visible range. It is worth pointing out that with the increase of pump power, the laser output power still increases linearly and does not tend to saturation. Thus, the power scaling is only limited by the currently available pump power. The output spectrum with a peak wavelength of 752.10 nm is shown in Figure 5(b). The FWHM of the spectrum at 752.10 nm is approximately 1.3 nm at an OSA resolution of 0.05 nm. As shown in Figure 5(c), the beam quality factors *M* ^2^ at ∼1 W of output power are $M_{x}^{2}$ = 3.2 and $M_{y}^{2}$ = 3.4 in the *x* and *y* directions, respectively. As presented in Figure 5(d), to investigate the power stability of the deep-red fiber laser, we measured every 5 min for 40 min at the laser output power of ∼1 W. As can be seen from the measured data, the root mean square error of the output power is around 10 mW, which corresponds to a relative power fluctuation of only 0.1%, demonstrating the uniform stability of the deep-red fiber laser.

### 3.2 Output characteristics of deep-red fiber lasers with different designs

**Figure 6:**
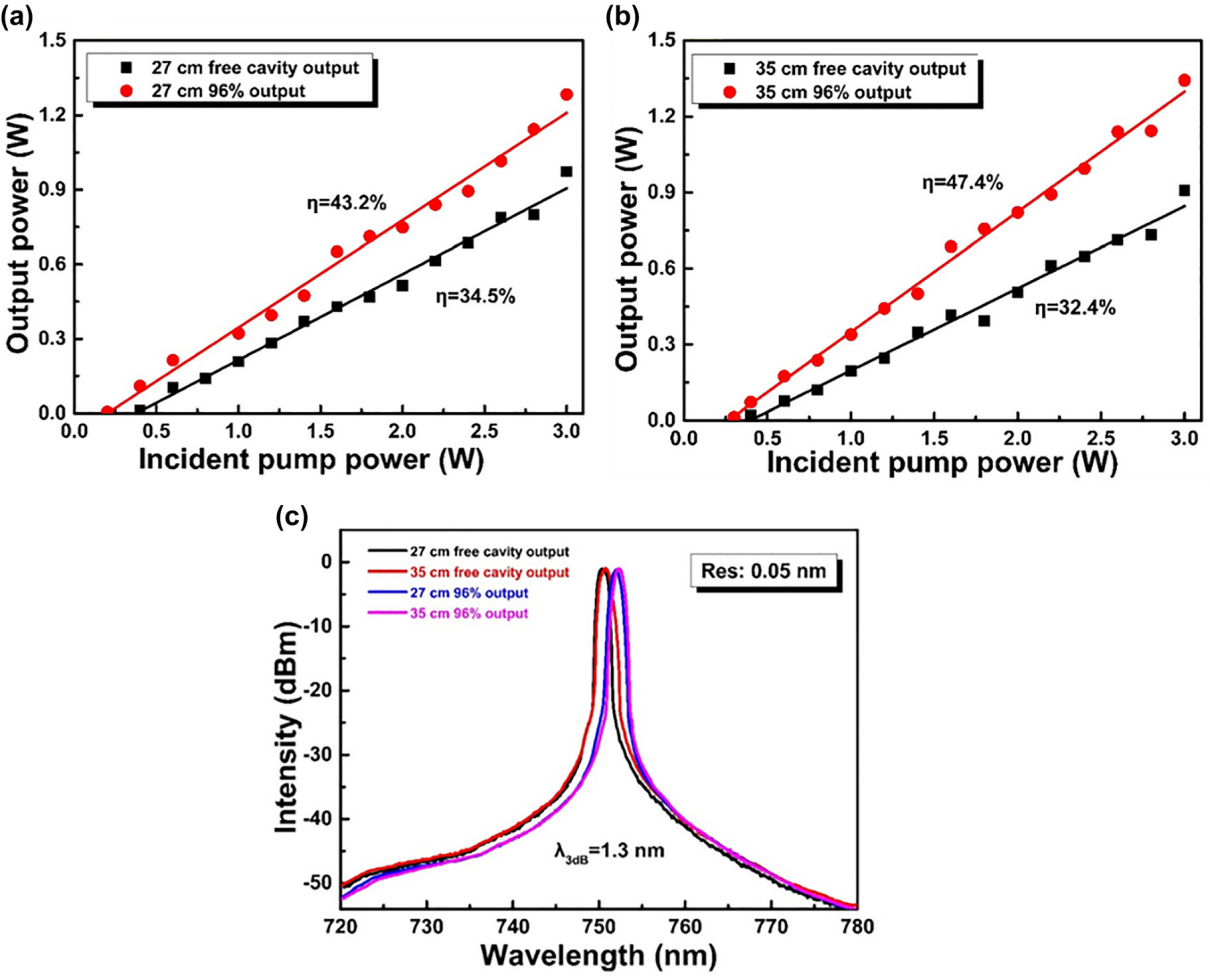
Deep-red laser output characteristics at different active fiber lengths (27 cm, 35 cm) and different output transmission.

Interestingly, due to the extremely strong ESA of ^5^I_7_ energy level, the laser resonator mirror *M* ^2^ is not necessary, and the deep-red laser output can be obtained by Fresnel reflection on the two end faces of the active fibers. Figure 6 shows the Ho^3+^-doped ZBLAN deep-red lasers output characteristics under different lengths and different transmissions. For an active fiber length of 27 cm, as is displayed in Figure 6(a), the maximum power of 96% output is 1.28 W with a threshold power of 0.20 W and the slope efficiency is 43.2%, while the maximum power of the double-terminal free cavity output is 0.97 W with a threshold power of 0.40 W and the slope efficiency is 34.5%. For another active fiber with a length of 35 cm shown in Figure 6(b), a maximum output power of 1.34 W was measured with a threshold power of 0.30 W, whose slope efficiency was 47.4% at a launched pump power of ∼3 W with the 96% laser output, while a maximum output power of 0.91 W is recorded with a threshold power of 0.42 W, and 32.4% slope efficiency with the double-terminal free cavity output. The output spectra are given in Figure 6(c), the wavelengths of the two laser cavities at the highest output power are around 750.50 nm and 752.20 nm, respectively. The results illustrate that despite the different gain fiber lengths, the output laser wavelengths are similar for the same cavity structure. The difference in wavelength between the two cavity structures can be attributed to the role of sublevels splitting in the lower energy level of the laser in the different pumping processes.

## 4 Conclusions

In summary, we systematically analyzed the different characteristics of blue LD downconversion and red solid-state upconversion pumping in Ho^3+^-doped fibers. We obtained CW deep-red fiber laser up to 1.64 W, using a green solid-state laser as the pump source. Furthermore, by optimizing the laser cavity design, watt-level output power of the free-cavity oscillation without additional cavity mirrors is attained, allowing for future exploration of deep-red laser applications. These investigations, in conjunction with the rapid development of commercial 532 nm solid-state lasers, will open up new and practical avenues for achieving single-wavelength high power and high efficiency operation in the visible spectral region.
